# Remission and Relapse Patterns of Type-II Diabetes Mellitus After Bariatric Surgery: A Retrospective Study at a Tertiary Care Center

**DOI:** 10.7759/cureus.111058

**Published:** 2026-06-17

**Authors:** Muhammad Bilawal Khan, Amina, Muhammad Nadir Shah, Siddique Ahmad

**Affiliations:** 1 General Surgery, Hayatabad Medical Complex Peshawar, Peshawar, PAK

**Keywords:** bariatric surgery and diabetes, diabetes mellitus type 2, hba1c, obesity and diabetes, type 2 diabetes remission

## Abstract

Background: Type-II diabetes mellitus (T2DM) is commonly associated with obesity and is generally treated with long-term medications. Bariatric surgery has demonstrated positive metabolic outcomes, including glycemic improvement and decreased use of antidiabetic medications. Remission can, however, not be permanent, and relapse of diabetes can occur in the follow-up period.

Objective: To assess T2DM remission, relapse, and postoperative glycemic outcomes after bariatric surgery in patients with obesity.

Methods: This retrospective observational study was conducted at the General Surgery Department, Hayatabad Medical Complex, Peshawar, Pakistan, between September 2022 and August 2025. A total of 93 patients who presented with obesity were included. The data on demographic characteristics, BMI, glycemic parameters, the use of antidiabetic medications, remission, and relapse were obtained from the hospital records. Follow-up was defined as the time from bariatric surgery to the last available postoperative clinic visit or laboratory assessment. Complete diabetes remission was defined as HbA1c <6.5% maintained for at least three months without glucose-lowering medication.

Results: The average age was 43.6 ± 9.8 years. The mean BMI was 44.2 ± 5.9 kg/m² prior to surgery and 33.1 ± 5.2 kg/m² at the most recent follow-up. The average HbA1c dropped to 6.4 ± 0.9% from 8.3 ± 1.4%, while fasting blood glucose decreased from 176.8 ± 42.5 mg/dL to 121.6 ± 28.4 mg/dL. A total diabetes remission was reached in 56 patients (60.2%), and partial improvement of glycemic levels in 21 patients (22.6%).

Conclusion: Bariatric surgery was associated with significant improvement in glycemic control, reduction in BMI, and decreased requirement for antidiabetic medications during available follow-up. Although a substantial proportion of patients achieved diabetes remission, relapse was observed in some cases, highlighting the need for long-term metabolic monitoring.

## Introduction

Type-II diabetes mellitus (T2DM) is characterized by insulin resistance, progressive β-cell dysfunction, and chronic hyperglycemia. The presence of excess adiposity contributes to insulin resistance, which makes obesity a significant risk factor for T2DM, increasing the metabolic demand on pancreatic β-cells [1,2]. Over time, this β-cell stress may lead to impaired insulin secretion and the development of T2DM. In many patients, conventional medical therapy improves glycemic control but does not fully address the underlying obesity-related metabolic dysfunction. Therefore, multiple medications, lifestyle modification, and ongoing monitoring are frequently necessary for long-term control of T2DM [3].

Bariatric surgery is becoming increasingly recognized as an important metabolic intervention among patients with obesity and T2DM [4]. The benefits of bariatric surgery go beyond weight loss. It enhances insulin sensitivity, lowers blood glucose levels, and decreases antidiabetic medication usage. Weight-dependent effects or hormonal and metabolic changes can occur after surgery [5]. Common procedures like sleeve gastrectomy and Roux-en-Y gastric bypass are increasingly acknowledged as effective ways for overweight and obese individuals to lose weight [6].

Following bariatric surgery, diabetes remission is a crucial clinical outcome. Remission is currently defined as having an HbA1c below 6.5% for three months straight without taking glucose-lowering drugs [7]. However, remission should not be interpreted as a permanent cure. Initial remission of hyperglycemia is followed by relapses in some patients, necessitating reintroduction of antidiabetic therapy. This relapse pattern is of clinical significance for patient counseling, long-term follow-up planning, and expectations for long-term surgical success [8].

Remission duration can be influenced by different factors such as pretreatment glycemic control, insulin dosage, diabetes duration before surgery, postoperative weight loss, and insulin deficiency [9]. Patients with shorter disease duration and low amount of medication use were more likely to achieve sustained remission, and others with long-standing diabetes might still be at risk for relapse following early improvement [10].

There is limited data locally on patterns of remission and relapse following bariatric surgery, especially in tertiary care surgical centers in Peshawar, Pakistan. Understanding these outcomes in the local population is important for improving patient selection, counseling, postoperative surveillance, and timely management of recurrent hyperglycemia.

## Materials and methods

This retrospective study was carried out in the General Surgery Department of Hayatabad Medical Complex, Peshawar, Pakistan, between September 2022 and August 2025. The Hayatabad Medical Complex's Institutional Review Board granted ethical approval under approval number 1179/HMC-QAD-F-00, dated August 8, 2022.

During the study period, 93 individuals with T2DM and obesity who had bariatric surgery were included. Patients of both genders who had a documented history of T2DM prior to surgery, who had undergone bariatric surgery, and had documentation for their glycemic status both before and after surgery were included in the study.

Individuals with gestational diabetes, type 1 diabetes, or secondary diabetes due to pancreatic or endocrine disorders, incomplete medical records, missing baseline HbA1c or fasting blood glucose, and those with inadequate postoperative follow-up data were excluded. Patients who had revisional bariatric surgery, surgery for primary indications other than metabolic, were also excluded.

The data were gathered from the hospital records, the operative notes, follow-up visits in the outpatient department, laboratory reports, and the records of the diabetes drugs used. A structured data collection pro forma was used to ensure uniformity. Age, gender, BMI, duration of T2DM, HbA1c at the time of surgery, fasting blood glucose at the time of surgery, use of diabetes medication, requirement for insulin, type of bariatric surgery performed, weight loss, HbA1c level after surgery, fasting blood glucose level after surgery, diabetes medication status after surgery, remission status after surgery, and duration of follow-up were all noted.

The postoperative glycemic outcomes were evaluated based on available follow-up visits throughout the study period. The follow-up period was defined as the time from the date of bariatric surgery to the last available postoperative clinic visit or laboratory assessment recorded before August 2025. HbA1c, fasting blood glucose, body weight, and use of antidiabetic medications were assessed at follow-up visits, where available. As this was a retrospective study, the duration of follow-up was not uniform for all patients and depended on the availability of outpatient follow-up visits and laboratory records. Therefore, the study mainly evaluated early and short- to intermediate-term postoperative glycemic outcomes rather than long-term durability of diabetes remission.

All patients underwent bariatric surgery under general anesthesia by a team of bariatric surgeons. The surgical procedure was determined based on clinical evaluation, the degree of obesity, metabolic profile, comorbidities, surgical fitness, and patient-surgeon interaction to identify the optimal procedure. Standard perioperative protocols were followed, including pre-anesthesia assessment, optimization of comorbid conditions, thromboprophylaxis where indicated, postoperative monitoring, dietary counseling, and scheduled follow-up.

T2DM was classified as a diagnosis of diabetes documented in medical records before bariatric surgery, the use of insulin or an oral hypoglycemic medication, or laboratory confirmation consistent with diabetes. Complete diabetes remission was not declared after a fixed number of years. It was defined as HbA1c <6.5% maintained for at least three months without the use of glucose-lowering medications. Partial glycemic improvement was defined as the reduction in HbA1c level and a decrease in the quantity or dosage of antidiabetic drugs postoperatively without meeting the criteria for complete remission. A diabetic relapse was defined as a return of glycemia to diabetic range (HbA1c ≥ 6.5%, fasting blood glucose ≥ 126) and/or the resumption of glucose-lowering therapy was restarted after documented diabetes remission.

The frequency of T2DM remission and relapse following initial remission was the primary outcome. Secondary outcomes included time to remission, time to relapse, changes in HbA1c and fasting blood glucose, change in BMI, reduction or discontinuation of antidiabetic medications, and factors associated with remission or relapse. The IBM SPSS Statistics for Windows, Version 26 (Released 2018; IBM Corp., Armonk, New York, United States) was used to analyze the data. The mean ± standard deviation was used to display continuous data such as age, BMI, duration of diabetes, HbA1C, fasting blood glucose, and follow-up period. For categorical variables like gender, surgery type, insulin use, remission status, and relapse status, frequencies and percentages were used. The normally distributed paired t-test and the non-normally distributed Wilcoxon signed-rank test were used to compare the preoperative and postoperative glycemic parameters. The chi-square test was used to look for relationships between categorical traits and remission or relapse. Statistical significance was defined as a p-value ≤0.05.

## Results

The final analysis comprised 93 patients in total. The baseline clinical and demographic information for the research population is shown in Table 1.

**Table 1:** Baseline demographic and clinical characteristics

Variable	Value
Age (mean±SD)	43.6 ± 9.8
Male, n(%)	39 (41.9%)
Female n(%)	54 (58.1%)
Weight (mean±SD)	119.4 ± 17.6
BMI (mean±SD)	44.2 ± 5.9
Duration of type-II diabetes median (IQR)	6.0 (3.0-9.0)
Hypertension n(%)	49 (52.7%)
Dyslipidemia n(%)	42 (45.2%)
Obstructive sleep apnea n(%)	18 (19.4%)
Preoperative HbA1c (mean±SD)	8.3 ± 1.4
Pre-op fasting blood glucose (mean±SD)	176.8 ± 42.5
Oral hypoglycemic agents only n(%)	41 (44.1%)
Insulin only n(%)	12 (12.9%)
Oral agents + insulin n(%)	19 (20.4%)
Lifestyle modification/no regular medication n(%)	21 (22.6%)

The most prevalent surgical procedure was sleeve gastrectomy, which was followed by mini gastric bypass (MGB) and Roux-en-Y gastric bypass (RYGB). The average hospital stay was 3.0 days, and the average surgical time was 92.4 ± 24.8 minutes (mean ± SD). Table 2 displays the outcomes of the procedures and the first postoperative phase.

**Table 2:** Operative and early postoperative characteristics RYGB: Roux-en-Y gastric bypass

Variable	Value, n = 93
Sleeve gastrectomy	58 (62.4%)
RYGB n(%)	23 (24.7%)
Mini gastric bypass n(%)	12 (12.9%)
Operative time, minutes, (mean ± SD)	92.4 ± 24.8
Length of stay, median (IQR)	3.0 (2.0-4.0)
Early post-op complications, n(%)	8 (8.6%)
Surgical site infection, n(%)	3 (3.2%)
Postoperative bleeding, n(%)	1 (1%)
Staple-line/anastomotic leak, n(%)	0 (0%)
30-day readmission, n(%)	2 (2.1%)
30-day mortality, n(%)	0 (0.0%)

Mean weight decreased from 119.4 ± 17.6 kg to 89.6 ± 15.8 kg, while the mean BMI decreased to 33.1 ± 5.2 kg/m² from 44.2 ± 5.9 kg/m². The mean fasting blood glucose decreased from 176.8 ± 42.5 mg/dL to 121.6 ± 28.4 mg/dL, while the mean HbA1c decreased from 8.3 ± 1.4% to 6.4 ± 0.9% (Table 3).

**Table 3:** Preoperative and postoperative changes in anthropometric and glycemic parameters Values are presented as mean ± SD. Preoperative and postoperative values were compared using the paired t-test. Test statistics are presented as t-values.

Parameter	Preoperative value	Last follow-up value	Mean change	Test statistic	p-value
Weight, kg, mean ± SD	119.4 ± 17.6	89.6 ± 15.8	-29.8	t = 12.15	<0.001
BMI, kg/m², mean ± SD	44.2 ± 5.9	33.1 ± 5.2	-11.1	t = 13.61	<0.001
HbA1c, %, mean ± SD	8.3 ± 1.4	6.4 ± 0.9	-1.9	t = 11.01	<0.001
Fasting blood glucose, mg/dL, mean ± SD	176.8 ± 42.5	121.6 ± 28.4	-55.2	t = 10.41	<0.001

Complete diabetes remission was achieved in 56 patients (60.2%), while 21 patients (22.6%) showed partial glycemic improvement. Remission patterns are presented in Table 4.

**Table 4:** Diabetes remission after bariatric surgery HbA1c <6.5% without the use of glucose-lowering drugs for a minimum of three months was considered complete remission.

Outcome	Value, n = 93
Complete diabetes remission, n (%)	56 (60.2%)
Partial glycemic improvement, n (%)	21 (22.6%)
No remission, n (%)	16 (17.2%)
Median time to remission, months, median (IQR)	7.0 (4.0-11.0)
Remission within 6 months, n (%)	24 (25.8%)
Remission within 12 months, n (%)	43 (46.2%)
Remission within 24 months, n (%)	52 (55.9%)
Remission by last follow-up, n (%)	56 (60.2%)

Among patients who achieved remission, 13 patients (23.2%) developed relapse during follow-up. A comparison between the maintained remission and relapse groups is shown in Table 5.

**Table 5:** Comparison of patients with maintained remission and relapse after initial remission Continuous variables are presented as mean ± SD or median (IQR), as appropriate. An independent-samples t-test was used for normally distributed continuous variables, the Mann-Whitney U test for non-normally distributed variables, and the chi-square test was used for categorical variables. Test statistics are reported as t-values, Z-values, or χ²-values, as appropriate.

Variable	Maintained remission, n = 43	Relapse, n = 13	Test statistic	p-value
Age, years, mean ± SD	41.8 ± 8.7	47.9 ± 9.6	t = 2.12	0.034
Female gender, n (%)	27 (62.8%)	6 (46.2%)	χ² = 1.14	0.285
Baseline BMI, kg/m², mean ± SD	45.1 ± 5.6	42.8 ± 6.2	t = 1.29	0.196
Duration of diabetes, years, median (IQR)	4.0 (2.0–7.0)	9.0 (6.0–12.0)	Z = 3.09	0.002
Baseline HbA1c, %, mean ± SD	7.9 ± 1.1	9.1 ± 1.5	t = 2.75	0.006
Preoperative insulin use, n (%)	8 (18.6%)	7 (53.8%)	χ² = 6.32	0.014
BMI reduction, kg/m², mean ± SD	12.4 ± 4.1	8.3 ± 3.7	t = 2.97	0.003
Median time to relapse, months, median (IQR)	-	18.0 (12.0–26.0)	-	-

Bariatric surgery was associated with a significant decrease in antidiabetic medication requirement, particularly insulin use. Medication changes are shown in Table 6.

**Table 6:** Change in antidiabetic medication status after bariatric surgery

Medication status	Preoperative, n (%)	Last follow-up, n (%)
No antidiabetic medication/lifestyle modification only	21 (22.6%)	58 (62.4%)
Oral hypoglycemic agents only	41 (44.1%)	27 (29.0%)
Insulin only	12 (12.9%)	3 (3.2%)
Oral agents plus insulin	19 (20.4%)	5 (5.4%)
Any insulin use	31 (33.3%)	8 (8.6%)
Discontinued medication among preoperatively medicated patients	-	37/72 (51.4%)
Insulin discontinued among preoperative insulin users	-	23/31 (74.2%)
Reduction in medication burden	-	24 (25.8%)
No meaningful change or escalation	-	11 (11.8%)

## Discussion

In the current study, 60.2% of patients achieved complete diabetes remission, and 22.6% of patients had partial glycemic improvement. The mean BMI decreased from 44.2 kg/m² to 33.1 kg/m², and the mean HbA1c decreased from 8.3% to 6.4%. These findings support the notion that bariatric surgery involves a metabolic intervention as well as a weight-loss procedure.

There is consistency between the remission rate of our study and the major bariatric surgery literature [11,12]. For patients undergoing RYGB, the diabetes remission rate among participants of the Purnell et al. study was 57% (seven years after surgery), a number near the rate of remission in our study [13]. The slight difference might be attributable to differences in the type of procedure, follow-up length, baseline glycemic control, and diabetes duration. We performed a preponderance of sleeve gastrectomies, which is in contrast to the stronger metabolic effects that are typically associated with bypass procedures. This could be the reason for the relatively high remission rates not being higher. Similarly, our results also support the findings of Courcoulas et al., who found that surgery was more likely to lead to durable remission if it occurred at an earlier stage in the disease and with a lower burden of diabetes medications [14].

The improvement in HbA1c and BMI in our study is also similar to the randomized evidence. In the STAMPEDE (Surgical Treatment and Medications Potentially Eradicate Diabetes Efficiently) study, the bariatric surgery group performed better in terms of long-term glycemic control and weight loss than the intensive medical therapy group [15]. The decrease in HbA1c from 8.3% to 6.4% in our cohort was a clinically significant decrease; however, the final mean HbA1c level still remained marginally higher than the remission threshold. This might be explained by the fact that some individuals needed insulin before surgery, follow-up was irregular, and patients with known diabetes were included. Similarly, the significant BMI loss achieved in our study corroborates the significance of weight loss in the improvement of insulin resistance and post-operative metabolic control [16].

Procedure type is an important consideration in interpreting our findings. Sleeve gastrectomy was the most common procedure in the current cohort. In the PCORNet Bariatric Study, RYGB performed better in terms of weight loss than sleeve gastrectomy, with somewhat greater diabetes remission, improved glycemic management, and fewer relapse occurrences [17]. Therefore, the high rate of sleeve gastrectomy in our study could have affected the remission and relapse pattern. However, sleeve gastrectomy still achieved some degree of metabolic improvement and is an effective procedure in clinical practice where patient preference, operative risk, and local expertise dictate the choice of procedure [18].

In our study, 23.2% had a relapse following initial remission. The clinical significance of this finding is that it suggests that the remission following bariatric surgery should not be viewed as a cure. Aminian et al. observed that, although there is a risk of late-relapsing diabetes following bariatric surgery, that is not a failure of treatment [19]. Instead, relapse can be due to the progressive loss in β-cell function, inadequate weight loss, weight regain, or worsening insulin resistance as time passes. We believe that our relapse rate is clinically reasonable, as these patients had higher baseline HbA1c, longer diabetes duration, more frequent insulin use before surgery, and less postoperative BMI loss.

Another significant outcome is the decrease in the need for antidiabetic drugs. Insulin use was reduced from 33.3% preoperatively to 8.6% at last follow-up, and 62.4% of patients stopped all glucose-lowering medicines in our study. This is similar to the Diabetes Surgery Study (DSS), which concluded that medical therapy alone is inferior to RYGB in terms of glycemic control and drug burden when paired with intensive medical therapy [20]. A reduction in medication use is clinically relevant as it can potentially enhance adherence, decrease treatment burden, and minimize medication-related adverse effects.

Overall, our findings suggest that bariatric surgery is an effective metabolic intervention for obese patients with T2DM in our setting. However, remission should be regarded as a dynamic metabolic state rather than a permanent cure. Regular follow-up assessments of HbA1C, fasting blood glucose, weight change, medication use, dietary habits, and weight regain should be carried out to ensure prompt detection of relapse and timely re-initiation of treatment.

This study's primary strength is the fact that it offers actual data from a Peshawar tertiary care surgical center, where local evidence regarding bariatric surgery and diabetes remission is limited. Another strength is the assessment of both remission and relapse, which gives a more clinically useful picture of postoperative metabolic outcomes. However, this study also has certain limitations, including a limited sample size, a single-center setting, a retrospective design, variable follow-up duration, a non-comprehensive analysis of C-peptide levels, and a lack of detailed evaluation of dietary compliance, physical activity, and weight regain after surgery. Due to the retrospective design and variable follow-up duration, this study mainly reflects early and short- to intermediate-term remission and relapse patterns. Therefore, late relapse and the long-term durability of diabetes remission could not be adequately assessed. Further prospective multicenter studies with standardized long-term follow-up are needed to confirm these findings and better evaluate the durability of diabetes remission after bariatric surgery.

## Conclusions

Patients with T2DM and obesity who underwent bariatric surgery showed significant improvement in glycemic control, reduction in BMI, and decreased requirement for antidiabetic medications during available follow-up. A substantial proportion of patients achieved diabetes remission; however, relapse was observed in some cases, indicating that remission should not be considered a permanent cure. Long-term monitoring with repeated assessment of body weight, medication use, fasting blood glucose, and HbA1c is essential for early detection of relapse and timely intervention.
